# Supplementary material for: High Variability Periods in the EEG Distinguish Cognitive Brain States
Source: Brain Sci. 2023 Oct 30;13(11):1528. doi: 10.3390/brainsci13111528 (PMC10669877; doi:10.3390/brainsci13111528)
Supplement: Supplementary file 1 [file brainsci-13-01528-s001.zip › brainsci-2606030-supplementary.pdf]

Suplementary Materials:

Supplementary Table S1: HVP Metrics at different window sizes

m	Overlap	Threshold	HVP-D	HVP-A	HVP-R	LVP-D	LVP-A	LVP-R	HVP-D/LVP-D
**3**	50%	10%	14.60	573	3.55	2.10	31	1.47	7.00
**5**	50%	10%	30.17	1425	1.68	5.05	99	0.49	6.05
**10**	50%	10%	42.97	2273	1.12	7.84	193	0.43	5.58
**15**	50%	10%	52.51	3047	0.88	10.10	264	0.40	5.21
**25**	50%	10%	64.22	3714	0.63	15.51	378	0.34	4.79
**30**	50%	10%	70.95	4105	0.55	23.60	212	0.20	4.51
**3**	50%	25%	7.48	332	5.58	2.94	51	3.44	2.55
**5**	50%	25%	16.49	864	2.43	6.74	146	1.16	2.46
**10**	50%	25%	26.99	1640	1.46	11.65	322	0.73	2.34
**15**	50%	25%	34.75	2109	1.05	14.97	455	0.58	2.37
**25**	50%	25%	47.09	2829	0.67	21.56	679	0.41	2.18
**30**	50%	25%	47.91	2810	0.58	22.71	752	0.41	2.11
**3**	50%	50%	4.56	254	5.70	4.85	100	5.18	0.94
**5**	50%	50%	10.06	638	2.33	10.75	266	2.12	0.96
**10**	50%	50%	16.58	1163	1.43	17.74	543	1.26	0.95
**15**	50%	50%	21.72	1497	1.02	23.20	774	0.91	0.95
**25**	50%	50%	31.27	2088	0.59	32.96	1201	0.53	0.97
**30**	50%	50%	35.05	2387	0.50	36.58	1396	0.47	0.97
**3**	10%	25%	14.68	639	2.66	5.49	99	1.06	2.69
**5**	10%	25%	22.46	1183	1.68	9.09	201	0.69	2.50
**10**	10%	25%	35.98	2088	0.99	15.90	453	0.45	2.34
**15**	10%	25%	47.67	2810	0.73	20.33	651	0.40	2.36
**25**	10%	25%	59.67	3255	0.46	28.33	861	0.36	2.24
**30**	10%	25%	62.24	3549	0.41	33.25	1162	0.34	2.06
**3**	25%	25%	10.92	478	3.69	4.19	73	1.77	2.61
**5**	25%	25%	19.59	1052	1.98	7.82	165	0.88	2.51
**10**	25%	25%	34.92	2037	1.05	15.23	423	0.47	2.33

**15**	25%	25%	42.99	2463	0.80	18.94	560	0.42	2.29
**25**	25%	25%	55.60	3132	0.50	25.88	871	0.36	2.36
**30**	25%	25%	57.30	3136	0.47	29.22	942	0.36	2.15

Supplementary Table S2: Statistical significance of differences between task conditions for two metrics and window sizes

HVP-D (window size = 10, overlap = 50%, threshold = 25%)

**Tukey's test**	EC	PC	EO	WM
Eyes Closed (EC)	NA	0.109	0.075	0.862
Pattern Completion (PC)	0.109	NA	1.000	0.015*
Eyes Open (EO)	0.075	1.000	NA	0.009*
Working Memory (WM)	0.862	0.015*	0.009*	NA

**Paired T-test**	EC	PC	EO	WM
Eyes Closed (EC)	NA	0.031*	0.054	0.205
Pattern Completion (PC)	0.031*	NA	0.899	0.001*
Eyes Open (EO)	0.054	0.899	NA	0.004*
Working Memory (WM)	0.205	0.001*	0.004*	NA

HVP-D (window size = 25, overlap = 50%, threshold = 25%)

**Tukey's test**	EC	PC	EO	WM
Eyes Closed (EC)	NA	0.016*	0.017*	0.845
Pattern Completion (PC)	0.016*	NA	1.000	0.001*
Eyes Open (EO)	0.017	1.000	NA	0.001*
Working Memory (WM)	0.845	0.001*	0.001*	NA

**Paired T-test**	EC	PC	EO	WM
Eyes Closed (EC)	NA	0.010*	0.030*	0.191
Pattern Completion (PC)	0.010*	NA	0.675	0.000*
Eyes Open (EO)	0.030*	0.675	NA	0.000*
Working Memory (WM)	0.191	0.000*	0.000*	NA

HVP-D/LVP-D (window size = 10, overlap = 50%, threshold = 25%)

**Tukey's test**	EC	PC	EO	WM
Eyes Closed (EC)	NA	0.016*	0.017*	0.845
Pattern Completion (PC)	0.016*	NA	1.000	0.001*
Eyes Open (EO)	0.017*	1.000	NA	0.001*
Working Memory (WM)	0.845	0.001*	0.001*	NA

**Paired T-test**	EC	PC	EO	WM
Eyes Closed (EC)	NA	0.316	0.480	0.002*
Pattern Completion (PC)	0.316	NA	0.286	0.032*
Eyes Open (EO)	0.480	0.286	NA	0.041*
Working Memory (WM)	0.002*	0.032*	0.041*	NA

HVP-D/LVP-D (window size = 25, overlap = 50%, threshold = 25%)

**Tukey's test**	EC	PC	EO	WM
Eyes Closed (EC)	NA	0.027	0.004	0.000
Pattern Completion (PC)	0.027	NA	0.919	0.283
Eyes Open (EO)	0.004	0.919	NA	0.665
Working Memory (WM)	0.000	0.283	0.665	NA

**Paired T-test**	EC	PC	EO	WM
Eyes Closed (EC)	NA	0.018*	0.010*	0.000*
Pattern Completion (PC)	0.018*	NA	0.351	0.059
Eyes Open (EO)	0.010*	0.351	NA	0.063
Working Memory (WM)	0.000*	0.059	0.063	NA

Supplementary Table S3: Correlation between HVP metrics at window size 9s and other metrics

HVP Metric	Measure	Correlation	*p-value*
Duration	Harmonic Regression Duration	0.18	0.37
Area	Harmonic Regression Duration	0.31	0.11
Rate	Harmonic Regression Duration	-0.22	0.27
HVP-D/LVP-D	Harmonic Regression Duration	-0.14	0.47
Duration	Lempel-Ziv Complexity	0.01	0.97
Area	Lempel-Ziv Complexity	0.06	0.76
Rate	Lempel-Ziv Complexity	0.01	0.96
HVP-D/LVP-D	Lempel-Ziv Complexity	0.18	0.37
Duration	Sample Entropy	-0.09	0.68
Area	Sample Entropy	-0.32	0.12
Rate	Sample Entropy	0.1	0.65
HVP-D/LVP-D	Sample Entropy	0.06	0.79
Duration	Waveform Complexity	0.17	0.42
Area	Waveform Complexity	-0.18	0.4
Rate	Waveform Complexity	-0.18	0.38
HVP-D/LVP-D	Waveform Complexity	0.39	0.053

Supplementary Table S4: Statistical significance of difference between EC and EO for different window sizes for HVP-D and Harmonic regression

Eyes Closed (EC) vs Eyes Open (EO) [Anova]		
**m**	**HVP-D *p-val***	**Harmonic *p-val***
3	6.6E-04	4.1E-01
5	1.2E-03	5.1E-01
10	1.9E-04	1.7E-01
15	4.8E-06	8.3E-03
25	7.0E-07	8.4E-03
30	2.0E-07	2.8E-02

Supplementary Table S5: Computation time per channel for each metric of variability

Metric	Computation Time per channel (s)
HVP metrics	0.11
Complexity	0.17
Harmonic Regression	53.69
Sample Entropy	7.41

Supplementary Table S6: Model performance for various metric combinations

EEG Metrics	Tasks	Sensitivity	Specificity	Precision	Recall	F1	Accuracy
HVP_A, HVP_D, HVP_R	EC vs EO	0.892	0.870	0.843	0.892	0.857	0.881
Sample Entropy, Theta/Beta, Alpha, Harmonic_D	EC vs EO	0.611	0.602	0.614	0.611	0.605	0.607
Sample Entropy, Theta/Beta, Alpha, Harmonic_D, HVP_A	EC vs EO	0.587	0.621	0.643	0.587	0.607	0.604
Sample Entropy, Theta/Beta, Alpha, Harmonic_D, HVP_A, HVP_D, HVP_R	EC vs EO	0.841	0.854	0.857	0.841	0.847	0.847
Sample Entropy, Theta/Beta, Alpha, Harmonic_D, HVP_D	EC vs EO	0.838	0.842	0.843	0.838	0.838	0.840
Sample Entropy, Theta/Beta, Alpha, Harmonic_D, HVP_R	EC vs EO	0.637	0.627	0.657	0.637	0.636	0.632
theta_by_Alpha, Alpha, HVP_A, HVP_D, HVP_R	EC vs EO	0.847	0.857	0.843	0.847	0.840	0.852
Theta/Beta, Alpha, Harmonic_D, HVP_A, HVP_D, HVP_R	EC vs EO	0.842	0.891	0.886	0.842	0.859	0.867
Theta, Alpha, Delta, Beta	EC vs EO	0.688	0.649	0.614	0.688	0.623	0.668
Theta, Alpha, Delta, Beta, HVP_A, HVP_D, HVP_R	EC vs EO	0.837	0.824	0.814	0.837	0.819	0.830

HVP_A, HVP_D, HVP_R	WM vs PC	0.718	0.808	0.767	0.718	0.727	0.763
Sample Entropy, Theta/Beta, Alpha, Harmonic_D	WM vs PC	0.705	0.786	0.783	0.705	0.732	0.745
Sample Entropy, Theta/Beta, Alpha, Harmonic_D, HVP_A	WM vs PC	0.609	0.745	0.717	0.609	0.649	0.677
Sample Entropy, Theta/Beta, Alpha, Harmonic_D, HVP_A, HVP_D, HVP_R	WM vs PC	0.742	0.822	0.800	0.742	0.761	0.782
Sample Entropy, Theta/Beta, Alpha, Harmonic_D, HVP_D	WM vs PC	0.767	0.875	0.867	0.767	0.807	0.821
Sample Entropy, Theta/Beta, Alpha, Harmonic_D, HVP_R	WM vs PC	0.682	0.795	0.767	0.682	0.715	0.738
theta_by_Alpha, Alpha, HVP_A, HVP_D, HVP_R	WM vs PC	0.802	0.904	0.883	0.802	0.833	0.853
Theta/Beta, Alpha, Harmonic_D, HVP_A, HVP_D, HVP_R	WM vs PC	0.747	0.861	0.850	0.747	0.788	0.804
Theta, Alpha, Delta, Beta	WM vs PC	0.700	0.735	0.700	0.700	0.689	0.717
Theta, Alpha, Delta, Beta, HVP_A, HVP_D, HVP_R	WM vs PC	0.771	0.863	0.850	0.771	0.801	0.817

HVP_A, HVP_D, HVP_R	WM vs EO	0.633	0.629	0.571	0.633	0.590	0.631
Sample Entropy, Theta/Beta, Alpha, Harmonic_D	WM vs EO	0.671	0.638	0.600	0.671	0.623	0.654
Sample Entropy, Theta/Beta, Alpha, Harmonic_D, HVP_A	WM vs EO	0.633	0.639	0.614	0.633	0.609	0.636
Sample Entropy, Theta/Beta, Alpha, Harmonic_D, HVP_A, HVP_D, HVP_R	WM vs EO	0.673	0.696	0.671	0.673	0.665	0.685
Sample Entropy, Theta/Beta, Alpha, Harmonic_D, HVP_D	WM vs EO	0.682	0.714	0.700	0.682	0.685	0.698
Sample Entropy, Theta/Beta, Alpha, Harmonic_D, HVP_R	WM vs EO	0.640	0.666	0.686	0.640	0.654	0.653
theta_by_Alpha, Alpha, HVP_A, HVP_D, HVP_R	WM vs EO	0.783	0.745	0.700	0.783	0.729	0.764
Theta/Beta, Alpha, Harmonic_D, HVP_A, HVP_D, HVP_R	WM vs EO	0.651	0.679	0.657	0.651	0.646	0.665
Theta, Alpha, Delta, Beta	WM vs EO	0.783	0.762	0.729	0.783	0.723	0.773
Theta, Alpha, Delta, Beta, HVP_A, HVP_D, HVP_R	WM vs EO	0.760	0.707	0.686	0.760	0.710	0.734

Supplementary Table S7: HVP metrics for different phases of anesthesia

Anesthesia Phase	m	HVP-D	SE (HVP-D)	HVP-A	SE (HVP-A)	HVP-R	SE (HVP-R)
Rest	3	7.59	0.56	1984.37	184.14	5.99	0.40
Lo-Anes	3	2.76	0.19	380.05	46.32	1.71	0.23
Dp-Anes	3	NA	NA	NA	NA	NA	NA
Recov	3	NA	NA	NA	NA	NA	NA
Rest	5	12.99	0.89	3998.69	426.16	3.16	0.15
Lo-Anes	5	5.03	0.17	727.69	77.28	1.13	0.16
Dp-Anes	5	NA	NA	NA	NA	NA	NA
Recov	5	NA	NA	NA	NA	NA	NA
